# Anatomic mapping of molecular subtypes in diffuse glioma

**DOI:** 10.1186/s12883-017-0961-8

**Published:** 2017-09-15

**Authors:** Qisheng Tang, Yuxi Lian, Jinhua Yu, Yuanyuan Wang, Zhifeng Shi, Liang Chen

**Affiliations:** 1 Department of Neurosurgery, Huashan Hospital, Fudan University, Shanghai, China; 2 Department of Electronic Engineering, Fudan University, Shanghai, China

**Keywords:** Glioma, Molecular diagnosis, Anatomic location

## Abstract

**Background:**

Tumor location served as an important prognostic factor in glioma patients was considered to postulate molecular features according to cell origin theory. However, anatomic distribution of unique molecular subtypes was not widely investigated. The relationship between molecular phenotype and histological subgroup were also vague based on tumor location. Our group focuses on the study of glioma anatomic location of distinctive molecular subgroups and histology subtypes, and explores the possibility of their consistency based on clinical background.

**Methods:**

We retrospectively reviewed 143 cases with both molecular information (IDH1/TERT/1p19q) and MRI images diagnosed as cerebral diffuse gliomas. The anatomic distribution was analyzed between distinctive molecular subgroups and its relationship with histological subtypes. The influence of tumor location, molecular stratification and histology diagnosis on survival outcome was investigated as well.

**Results:**

Anatomic locations of cerebral diffuse glioma indicate varied clinical outcome. Based on that, it can be stratified into five principal molecular subgroups according to IDH1/TERT/1p19q status. Triple-positive (IDH1 and TERT mutation with 1p19q codeletion) glioma tended to be oligodendroglioma present with much better clinical outcome compared to TERT mutation only group who is glioblastoma inclined (median overall survival 39 months VS 18 months). Five molecular subgroups were demonstrated with distinctive locational distribution. This kind of anatomic feature is consistent with its corresponding histological subtypes.

**Discussion:**

Each molecular subgroup in glioma has unique anatomic location which indicates distinctive clinical outcome. Molecular diagnosis can be served as perfect complementary tool for the precise diagnosis. Integration of histomolecular diagnosis will be much more helpful in routine clinical practice in the future.

## Background

Glioma is the most common malignant brain tumor with heterogeneous growth pattern which can be found in different cerebral lobes [1, 2]. This kind of locational variety has been demonstrated to be of great importance in patient diagnosis and prognosis which can reflect the tumor cells origin as well [3]. Many studies have been performed to prove relationship between molecular biomarkers and tumor location. [2, 4–6]. Recently, new WHO classification of cerebral diffuse gliomas was revised with complementary of three molecular biomarkers (IDH1/1p19q/H3F3A) integrated into comprehensive pathological diagnosis [7]. It demonstrated that glioma-related biomarkers have been playing much more important role in precise medicine. Robert B.Jenkins et al. has successfully used three major biomarkers to classify glioma into five principal molecular subsets represent distinctive clinical significance and germline variants. This finding hallmarked the development of molecular pathology in glioma which was published on New England Journal of Medicine [8]. In our study, we plan to use same stratification system in our patients cohort and delineate tumor locational tendency within different molecular subtypes. Furthermore, we will explore the consistency of tumor location between histological subtypes and their molecular counterparts to identify the complementary effect of molecular diagnosis in cerebral diffuse glioma, especially in outcome prediction.

## Methods

### Patients and tissue samples

We searched for Molecular Database and Image Bank in Department of Neurosurgery, Huashan Hospital and retrospectively selected 143 glioma samples for further study. H&E slides of all cases were reviewed by 2 individual neuropathologists to confirm the diagnosis of glioma according to WHO 2016 brain tumor guideline. Every patient has molecular diagnostic information. 140 out of 143 patients got complete follow-up. This research work was approved by Ethic Committee of Huashan Hospital and informed consents signed. Patients characteristic are listed in Table 1.

**Table 1:** Characteristics of all patients

Characteristic	Total number	Sex		Age		
	143	Male	Female	0–35	36–60	>60
Molecular subtype						
Triple-positive	41 (29%)	23	18	9	32	0
TERT and IDH mutation	7 (5%)	3	4	4	3	0
IDH mutation only	37 (26%)	25	12	19	18	0
Triple-negative	27 (19%)	14	13	7	17	3
TERT mutation only	30 (21%)	21	9	5	13	12
Other	1 (1%)	1	0	1	0	0
WHO grade						
Grade II	82 (57%)	45	37	34	45	3
Grade III	27 (19%)	17	10	6	21	0
Grade IV	34 (24%)	25	9	5	17	12
Pathological Diagnosis						
Astrocytoma	69 (48%)	43	26	29	37	3
Oligodendroglioma	40 (28%)	19	21	11	29	0
Glioblastoma	34 (24%)	25	9	5	17	12

### Molecular profiles of IDH1/TERT/1p19q

Paraffin blocks of each case were prepared. Four 4um slides and six 10um slides were sectioned. DNA extraction was performed by commercial DNA extraction kit (Qiagen, Shanghai) using 10um slides. IDH1 and TERT mutational analysis were done by Sanger Sequencing with method reported previously [9]. The status of 1p19q was determined by FISH (fluorescence in situ hybridization). A case of 50% tumor cells present with reference probe signal ratio to target probe signal more than 2:1 was considered 1p19q codeletion, otherwise we called 1p19q intact [10]. Molecular information will be integrated into regular pathological diagnosis.

### Tumor location analysis

Image segmentation is an important pre-processing step for location analysis. Convolutional neural network (CNN) was proved to be an effective method for medical image segmentation [11]. In our research, an approach based on CNN was adopted to extract brain tumors on MR images, which got satisfactory performance in the Brain Tumor Segmentation Challenge 2013 and 2015. (http://www.braintumorsegmentation.org/).

In order to study the location features in same coordinate system, the segmentation results were registered to MNI152 (Montreal Neurological Institute (MNI)) brain atlas [12]. A research platform also provided by MNI, SPM12, was used to accomplish this procedure. Both MNI152 and SPM12 were widely used in brain tumor registration [13].

### Statistical analysis

Correlation coefficient was calculated to figure out the location relation between specific histology stratification and molecular phenotype. IBM SPSS statistic 20.0 software (SPSS, Chicago, IL, USA) was chosen as the analysis tool. A strong association would be found out with *r* value closed to 1. Median overall survival time (OS) was defined as the duration from the diagnosis and death or the last follow-up. Kaplan-Meier method was used to draw survival curve and analyzed by Log-rank test.

## Results

### The molecular combination of IDH1/TERT/1p19q has unique distribution among distinctive histological subtypes in glioma

**Fig. 1:**
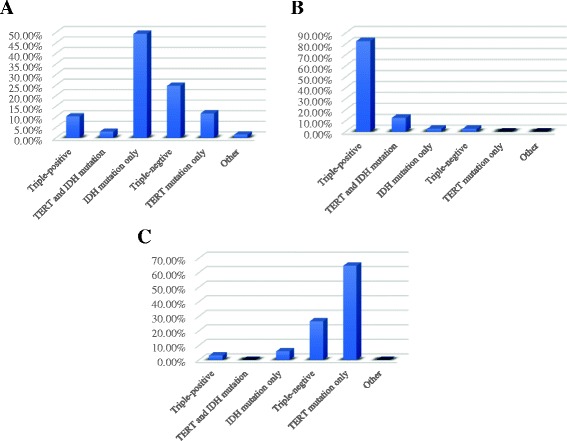
Distribution of IDH1/TERT/1p19q among distinctive histological subtypes. **a** astrocytoma, (**b**)oligodendroglioma, (**c**)glioblastoma

The information of histology diagnosis and WHO grade among 143 glioma patients can been found in Table 1. Accordance with WHO 2016 instruction, oligoastrocytoma was subdivided into oligodendroglioma or astrocytoma according to 1p19q status [7]. By using panel of IDH1/TERT/1p19q, we divided the whole case cohort into 5 molecular subgroups according to NEJM paper [8]. There are 41 Triple-positive and 27 triple-negative tumors. Proportion of TERT and IDH1 mutation, IDH1 mutation only and TERT mutation only subgroup accounts for 26%, 19% and 21% respectively. Similar to previous studies, IDH1 mutation only tumors were more likely to be seen in astrocytoma with the ratio of 49.3%, while 82.5% triple-positive gliomas belong to oligodendroglioma. 64.7% glioblastomas have TERT mutation only. (Fig. 1).

### Different survival outcomes among distinctive molecular subgroups based on IDH1/TERT/1p19q classification system

**Fig. 2:**
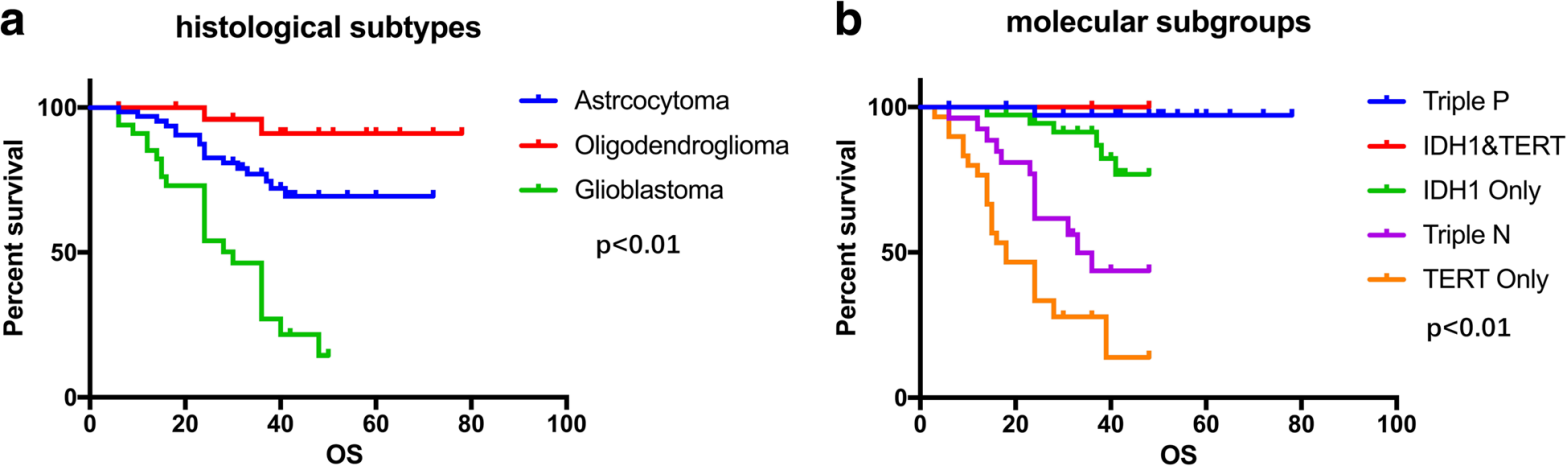
Median overall survival time for different histological subtypes (**a**) and molecular subgroups (**b**)

We have completely follow-up in 140 patients, among whom 43 patients were dead and the other patients were still alive. Patients diagnosed as oligodendroglioma have the best clinical outcome with median overall survival 37.9 months (*P* < 0.01). Median overall survival are 33 months for astrocytoma and 20.5 months for glioblastoma (Fig. 2a). Regarding to molecular stratification, patients in triple-positive subgroup have the best survival outcome with 39 months of median overall survival compared to IDH1/TERT mutation subgroup (36.9 months), IDH1 mutation only subgroup (34 months), triple-negative subgroup (27.6 months) and TERT mutation only subgroup (19.9 months) (*P* < 0.0001) (Fig. 2b).

### Patients with different anatomic position have unique survival outcome

**Fig. 3:**
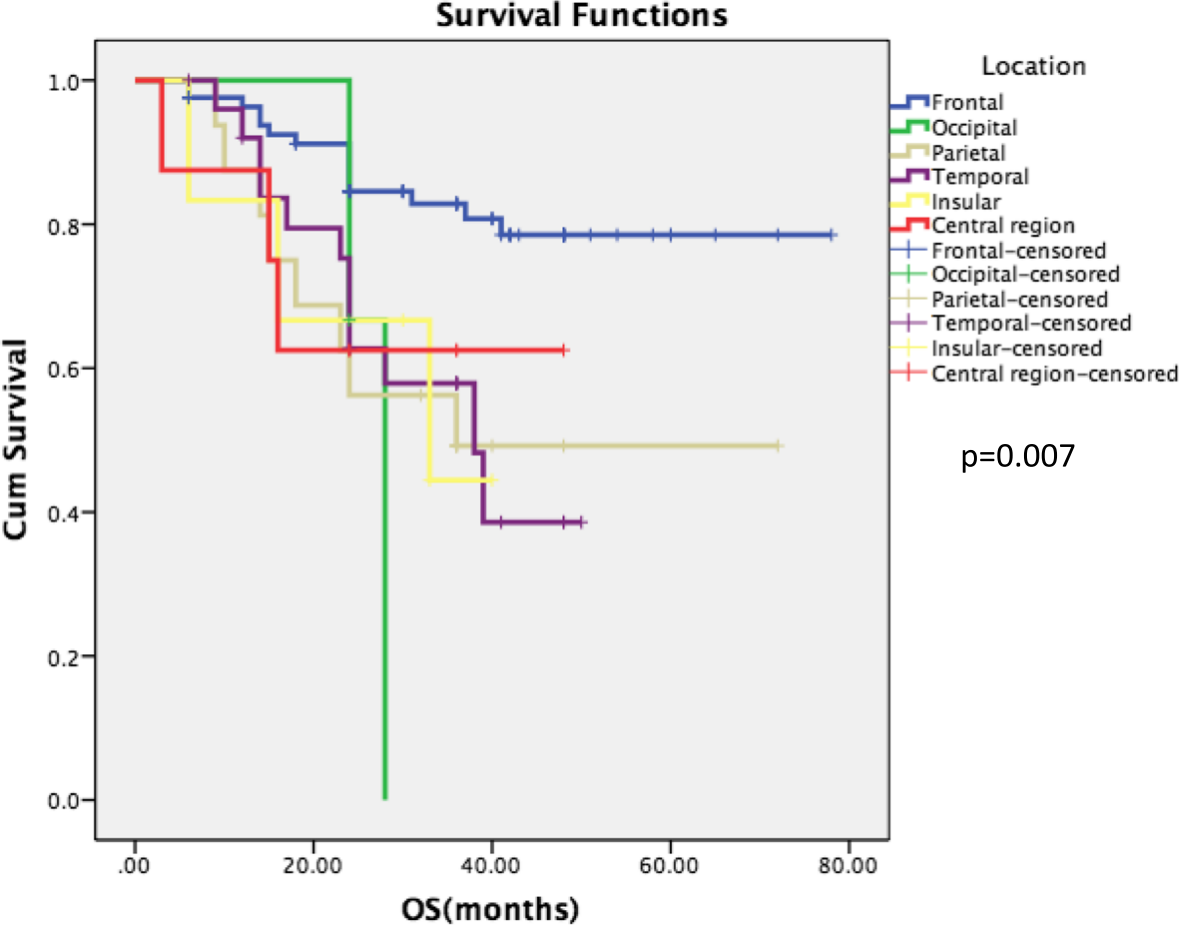
Survival outcome among different location subpopulations

Previous to studying anatomic preference to special molecular subgroups, we analyzed survival outcome between different tumor location. Herein, we found tumor located in frontal lobe indicated longer overall survival time of 66.1 months compared to tumors located in other cerebral regions (*P* < 0.01). Tumors located in hemisphere demonstrate better clinical outcome than those in central region even though no significance exists (Fig. 3).

### Locational pattern is different among distinctive molecular subgroups

**Fig. 4:**
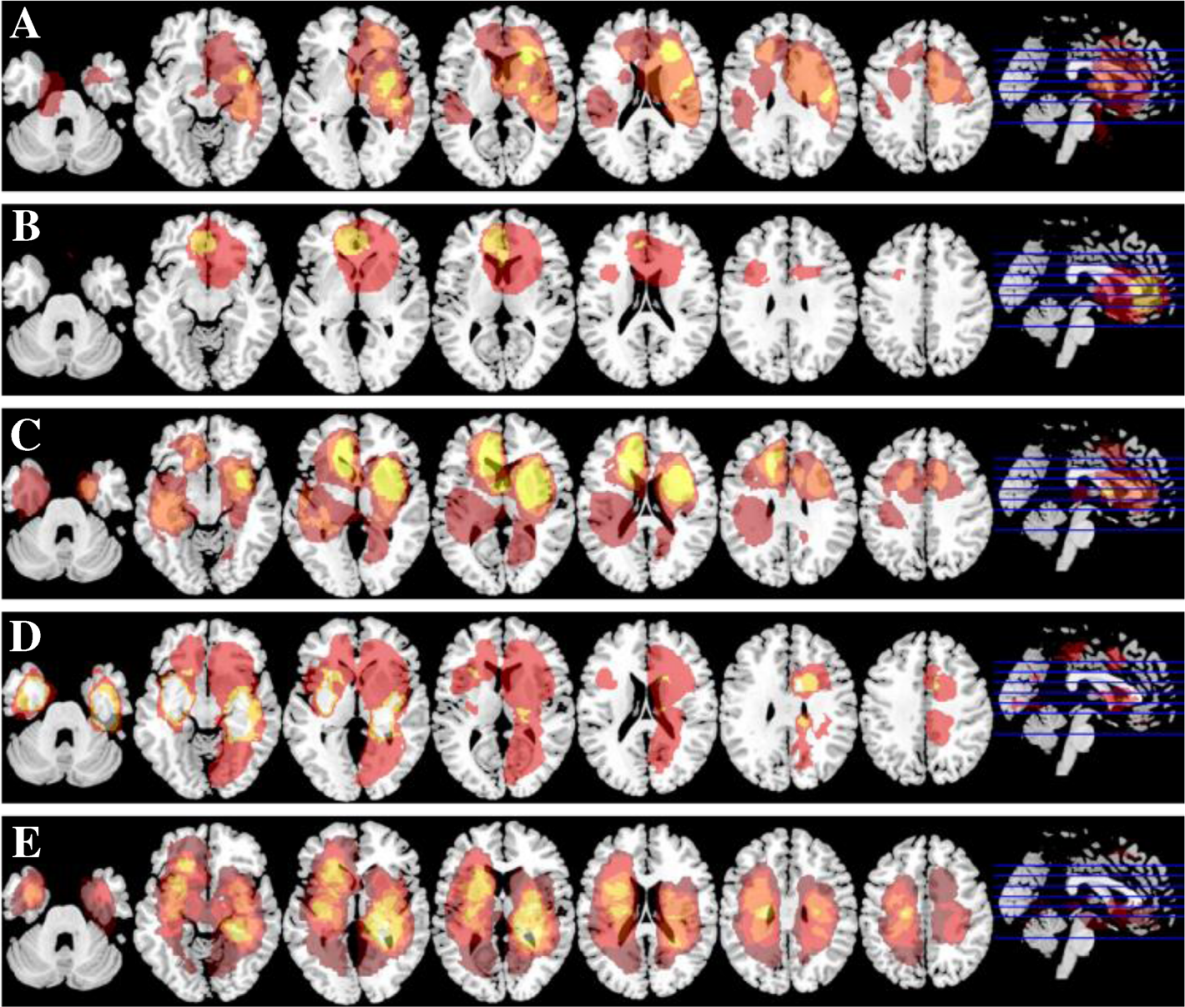
Distribution of tumor location according to IDH1/TERT/1p19q stratification regimen. **a** Triple-positive, (**b**) TERT and IDH mutation, (**c**) IDH mutation only, (**d**)Triple-negative, (**e**)TERT mutation only

For triple-positive gliomas, tumor location tends to aggregate in bilateral frontal lobes. On the contrary, triple-negative tumors were more likely to locate in bilateral basal ganglia regions. In spite of that, IDH1/TERT mutation subgroup inclined to grow in left frontal lobe close to midline region. IDH1 mutation only subgroup was commonly seen in left frontal lobe and bilateral insular lobes. TERT mutation glioma apparently present with non-midline distribution while sitting in right frontal-insular lobe and left basal ganglia region. Meanwhile, TERT mutation only glioma has deep-seated location than triple-positive cases (Fig. 4).

### Tumor location remains consistently between histological subtype and its corresponding molecular subgroup

**Fig. 5:**
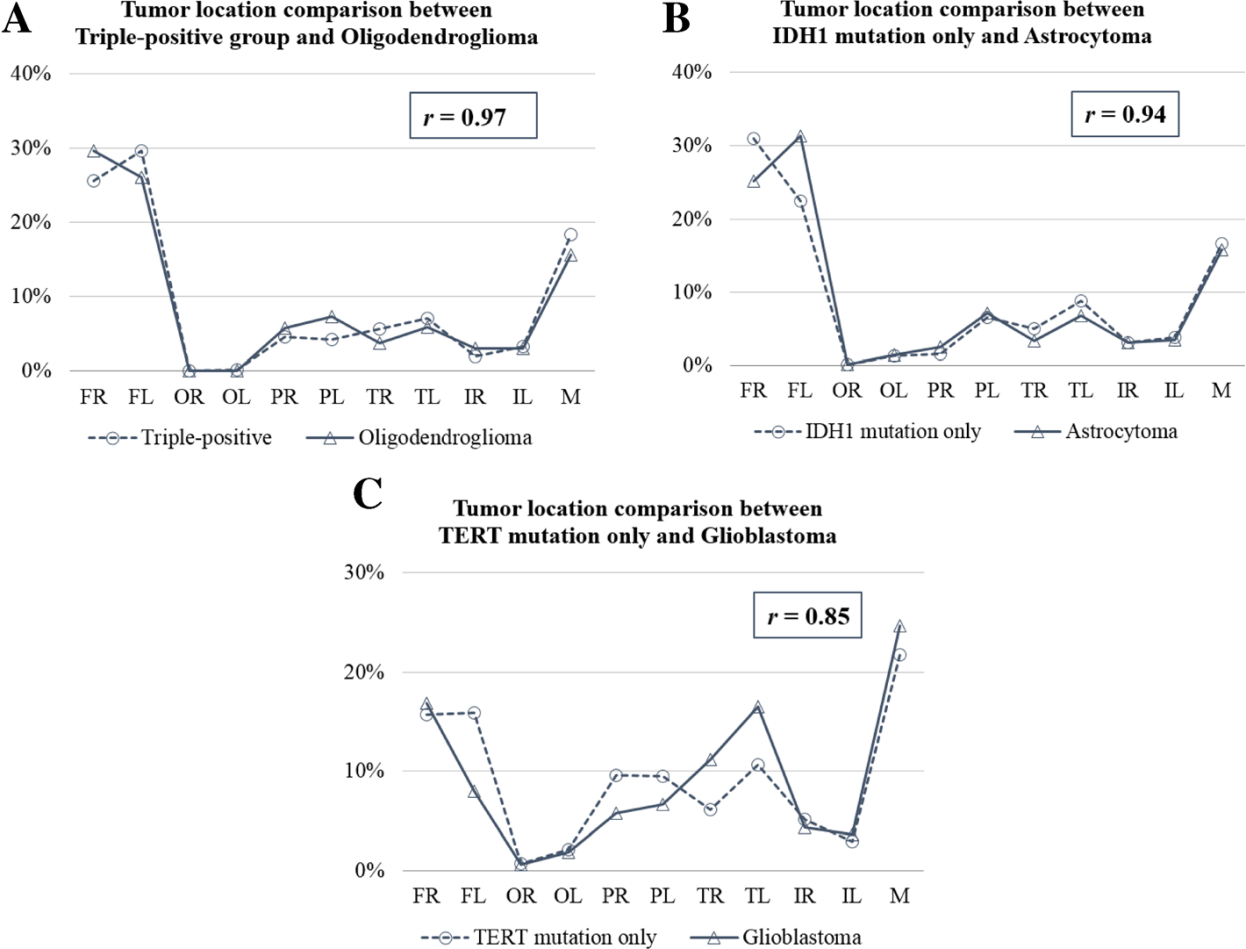
Tumor location correlation analysis between histological subtype and its corresponding molecular subgroup with (**a**) triple-positive group vs. oligodendroglioma, (**b**) IDH1 mutation only vs. astrocytoma, (**c**) TERT mutation only vs. glioblastoma

Thus glioma histology stratification strongly associated with molecular phenotype, we investigate whether anatomic distribution remains consistent within these two classification systems. We compared triple-positive samples with oligodendroglioma, IDH1 mutation only subgroup with astrocytoma and TERT mutation only tumors with glioblastoma since these genetic events highly represent histological diagnosis. It’s interested to find out that the tumor location and growth pattern is quite similar to each other based on MR images. The location correlation coefficients are 0.97, 0.94 and 0.85 accordingly (Fig. 5a–c).

### Dissimilarity in molecular background results in different tumor location within mixed diffuse glioma

**Fig. 6:**
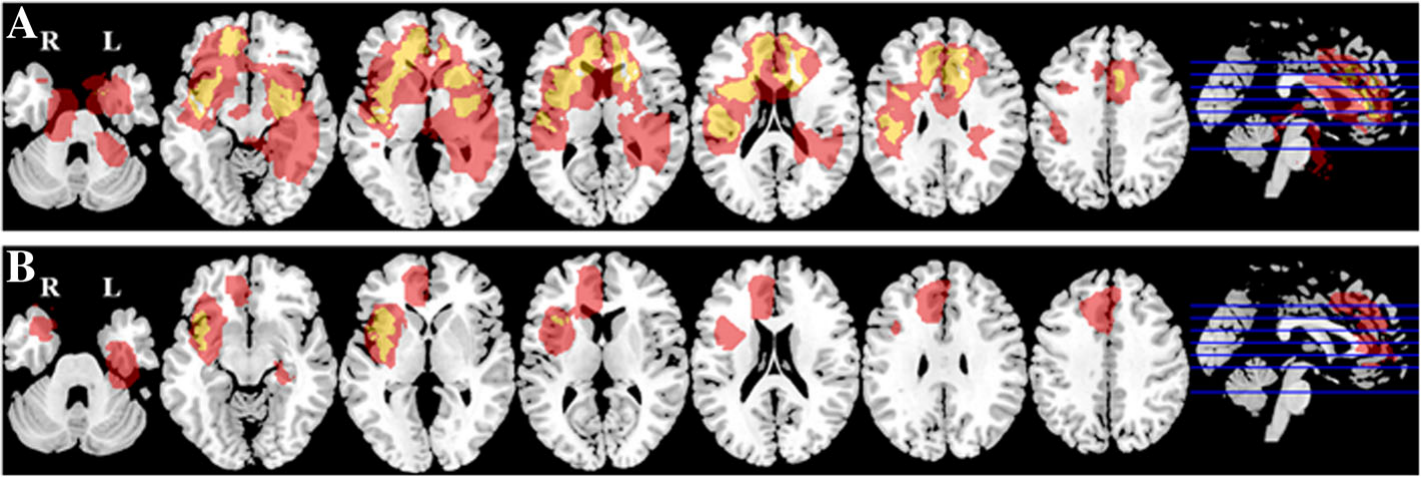
1p19q status of oligoastrocytoma decide tumor location, (**a**) Oligoastrocytoma with1p19q codeletion, (**b**) Oligoastrocytoma with 1p19q retain

In 2016 revised WHO diffuse glioma classification, the diagnosis of oligoastrocytoma was gone due to 1p19q can help clearly subdivide tumor into oligo-lineage or astroglial family. We have 14 oligoastrocytoma cases, 10 of them showed 1p19q codeletion with tumor likely to locate in bilateral frontal lobe which is similar to oligodendroglioma. On the other hand, tumor with 1p19q intact tends to grow in left insular lobe where is commonly seated by astrocytoma (Fig. 6).

## Discussion

Cerebral diffuse glioma is a biological heterogeneous tumor [1]. Patient clinical outcome was affected by many factors including age, anatomic location, tumor size, extent of resection, genetic alteration [14]. Among them, anatomic location plays a crucial role not only for prognosis prediction but also for treatment strategy. It was widely acknowledged that prognosis is poor in midline glioma than non-midline tumor [10]. Regarding to hemispheric glioma, patient with tumor located in frontal lobe tends to be younger, IDH1 mutation and longer survival time [15]. This conclusion is accordance with our result. Furthermore, in our cohort, occipital lobe glioma implied negative impact on clinical outcome which is similar to Liu et al. [16]. The reason might be larger tumor size commonly seen in this area. Meanwhile, anatomic location somehow determines the extent of resection, for example, midline or deep-seated glioma is hard to get gross total resection due to preservation of functional structure or complex surgical corridor [17]. On the contrary, non-eloquent area tumor, especially superficial to the cortex is amendable to completely remove. For the same reason, longer survival time is strongly associated with gross total resection [18]. On the other hand, based on huge amounts of exploration of glioma genetic alteration in the recent years, a panel of classic biomarkers begins to exert great impact on glioma precise diagnosis and prognostic assessment [1]. IDH1/1p19q/H3F3A are the representatives introduced into newly revised 2016 WHO glioma guideline [7]. In our previous study, 4 biomarkers were used to stratify lower grade glioma into 4 subgroups predicting better clinical outcome than the roles of histological diagnosis and WHO grade [19]. The current findings firmly validate the great prognostic value of biomarkers in glioma. Similar to our findings, Jenkins et al. used a genetic combination of IDH1/1p19q/TERT to classify glioma into 5 subpopulations with unique clinical features and germline variants respectively which is highly recognized in the world [8]. We referred to this 3-biomarkers scheme in our study, and drew the same conclusion Triple-positive and IDH1/TERT double-mutation cases are more likely to be oligo-lineage. IDH1 mutation only cases are astrocytoma with maximal possibility. IDH1 wild type and TERT mutation tumors are commonly seen in glioblastoma. According to this scheme, survival outcome in our patient cohort is distinctive among all the subgroups. All these data demonstrate integration of molecular and histology diagnosis being helpful in prognostic and predictive value for glioma patients. Nevertheless, the perfect integration of these two systems still calls for huge efforts like large cohort clinical validation on many aspects, such as image features. Thus, we hypothesized anatomic location, genetic biomarkers and histology diagnosis are highly correlated and intertwined.

In order to verify our hypothesis, we tried to study the interconnection between anatomic location and genetic biomarkers in our patient cohort. Beforehand, many papers published have put forward the cell origin theory underlying possible relationship between these two factors [20, 21]. Many groups have successfully developed computational methods to predict glioma genetic alterations based on location features [18, 22, 23]. For example, IDH1 mutation was commonly seen in left frontal lobe, where TERT mutation only exists as well[2, 5, 23]. Such investigations were performed in the context of MGMT and TP53[24, 25]. In our study, we used a panel composed of IDH1/1p19q/TERT which is worldwide recognized in the precise diagnosis of glioma to demonstrate the anatomic distribution of different molecular subsets. Our data showed similar results to previous research works such as IDH1 mutation prefers to localize in left frontal lobe[2, 15]. Interestingly, we also found that triple-positive tumor located more superficial to cortex than TERT mutation only tumor. This finding may explain the differences in survival outcome and extent of resection. In spite of that,. highly consistency of location feature was observed between histological subpopulations and its corresponding molecular counterparts. For example, triple-positive tumor appears to have similar anatomic location with oligodendrogliomas. That means the axis of molecule-cell-tissue depicts the growth pattern of glioma which is additional evidence supporting the cell origin theory. Another interesting finding is that 1p19q codeletion oligoastrocytoma possessing different anatomic location with 1p19q intact oligoastrocytoma which is supportive to new 2016 WHO classification that the diagnosis of oligoastrocytoma is eliminated [7]. Since now, the diagnosis of oligoastrocytoma converts to either oligodendroglioma or astrocytoma according to molecular biomarkers [26]. These findings demonstrate definite molecular feature restricted to precise histological diagnosis. It strongly proved that molecular diagnosis can help clinicians make exactly right diagnostic decision facilitating to tailor personalized treatment.

On the other aspect, methods by using MR images to predict molecular biomarkers are popular recently, which was so-called Radiomics study. Ellingson et al. compared tumor volume ratio of T2 hyperintensity to contrast enhancement and central necrosis to differentiate mesechymal and non-mesenchymal molecular subtype in glioblastoma [27]. His research team also used perfusion and diffusion MRI signatures to successfully stratify lower grade glioma into three subpopulations as IDH1 mut/1p19q codel, IDH1 mut/1p19q non-condel and IDH1 wt [28]. MRS is another popular detectable technology to realize Radiomics study due to unique metabolic features inside glioma. It has been widely applied to predict IDH1 mutational status and medulloblastoma subgrouping [29, 30]. Compared to these methods, our team used anatomic location as basic tumor feature to predict biomarkers like IDH1/1p19q/TERT, which is more simple, cost effective and visualized. The raw materials we need are only T2 flair and T1 contrast MR images without sophisticated computation process. However, our method has its own limitations, like rough estimation accuracy. In general, our team illustrated a simple method to predict molecular biomarkers and reveal anatomic location among different molecular subgroups which offered an alternative in Radiomics study.

## Conclusion

Although molecular biomarkers are getting involved in routine pathological diagnosis of cerebral diffuse gliomas, more evidence should be provided to validate perfect match between molecular subtypes and classic histological diagnosis. Our study showed distinct anatomic distribution among different molecular phenotypes which is consistent with corresponding histological subtypes. Integration of molecular biomarkers with histology diagnosis will not only contribute to precise diagnosis but also predict patient clinical outcome. This kind of pathological diagnosis system was highly recommended in future clinical practice. Moreover, we developed a simple and cost-effective method to predict biomarkers which was supposed to be widely used in Radiomics study in glioma.

## Abbreviations

CNN: Convolutional neural network
MNI: Montreal Neurological Institute
